# Impacts of Rock Mineral and Traditional Phosphate Fertilizers on Mycorrhizal Communities in Pasture Plants

**DOI:** 10.3390/microorganisms11041051

**Published:** 2023-04-17

**Authors:** Ahmed R. Alsharmani, Zakaria M. Solaiman, Matthias Leopold, Lynette K. Abbott, Bede S. Mickan

**Affiliations:** 1 School of Agriculture and Environment, and UWA Institute of Agriculture, The University of Western Australia, Perth, WA 6009, Australia; ahmadrs2012@gmail.com (A.R.A.); zakaria.solaiman@uwa.edu.au (Z.M.S.);; 2 College of Science, University of Kufa, Najaf 54001, Iraq

**Keywords:** arbuscular mycorrhizal fungi, grasslands, nutrient cycling, phosphorus, roots, soil biology, rhizosphere

## Abstract

Intensive fertilizer use can constrain contributions from soil biological processes in pastures, including those associated with arbuscular mycorrhizal (AM) fungi. We evaluated the effect of fertilizers of different P solubility on the colonization of the roots of two common pasture plants by a community of AM fungi in a pasture soil. The treatments were a rock mineral fertilizer, a chemical fertilizer and a microbial inoculant. Subterranean clover and annual ryegrass were grown in pots for 10 weeks. Both fertilizers reduced the proportion and length of roots colonized by naturally occurring AM fungi. However, by 10 weeks, there was a much greater length of mycorrhizal root for annual ryegrass than for subterranean clover. The relative abundance of mycorrhizal fungi in the families Glomeraceae and Acaulosporaceae in roots was not affected by the form of fertilizer, but diversity indices of AM fungi in roots were altered. The chemical fertilizer had a greater negative effect on AM fungal diversity indices in the annual ryegrass roots compared with the subterranean clover roots. The reduction in OTU richness of AM fungi with fertilizer application corresponded with reduced soil pH. Differential effects of P fertilizers on naturally occurring AM fungi in this agricultural soil have the potential to influence the efficacy of P fertilizer use and dominance of plant species in grasslands.

## 1. Introduction

In grassland systems, the application of fertilizers is the most common management practice for promoting pasture productivity. Different kinds of fertilizers, including organic P-based fertilizers, are applied to regulate the P status of grassland soil [1,2]. Although the use of conventional P fertilizers can promote rapid plant growth, they are expensive, especially in developing regions [3], and may have adverse influences on soil microbial communities or on the environment if applied beyond plant requirements under the prevailing soil conditions [4,5,6]. Thus, alternative fertilizers such as controlled- or slow-released fertilizers have been used to minimize negative consequences of supplying unnecessarily high levels of soluble P [7,8].

Phosphate rocks have been incorporated as major components of P in some slow- or controlled-release fertilizers [9,10,11]. P solubilizing microorganisms have been considered a low-cost and low-energy mechanism to promote the agronomic effectiveness of phosphate rock fertilizers [12,13] but their benefits may be limited [14]. An extension of this approach is the use of phosphate rocks in combination with P solubilizing microorganisms and arbuscular mycorrhizal (AM) fungi [15].

The application of P fertilizers to soil can alter soil microbial communities in a range of soil health-related processes and this is well documented [16,17]. An example is reduced soil bacterial alpha diversity indices following the application of fertilizers differing in P solubility which increased growth of two pasture species in an agricultural soil from Pingelly in south-western Australia [18]. This fertilizer effect occurred in parallel with reduced soil pH for subterranean clover but not for annual ryegrass. A microbial inoculant applied in the same study did not affect shoot growth 10 weeks after sowing, but rhizosphere bacterial beta diversity indices differed markedly between the same two plant species [18].

Arbuscular mycorrhizal (AM) fungi are common in grassland soils and colonize most pasture plant species [19,20]. This mycorrhizal symbiosis can facilitate the plant uptake of nutrients, especially P and to a lesser extent N, in addition to other soil resources such as water, and contribute to other soil health benefits [21,22,23]. Nutrient enrichment of grasslands, especially with P and N, can significantly affect root growth, and in turn, plant productivity. Furthermore, factors that influence the growth of roots can alter the extent to which mycorrhizas are formed. Grazing can alter the carbon availability in roots for the growth of AM hyphae [19], and mycorrhizal responsiveness of pasture plant species depends on root architecture [24]. The type, rate and duration of fertilization can significantly affect the abundance, composition and diversity of AM fungi [25]. Plant nutritional contributions of AM fungi associated with P fertilizers depend on the extent of hyphal extension beyond the root surface into un-depleted P zones [24,26]. Furthermore, as AM fungi differ in their capacity to colonize roots and form hyphal networks in soil [27], changes in fungal dominance could be expected to influence mycorrhizal contributions to plant nutrition and to soil health more generally [22].

The colonization of roots by AM fungi is most commonly assessed based on the proportion of the root system colonized [28]. However, the total biomass of AM fungi in a soil system depends on both the extent of the root system colonized (proportion) and the mass or length of root colonized [28]. In a general way, it is possible that having a greater proportion of the root system colonized by AM fungi is necessary for nutritional benefits and that a greater mass of colonized roots is necessary to achieve benefits to soil structure [19]. However, there are no direct relationships between these traits and function, which vary with the ecological situation [22]. In addition to quantifying the proportion or mass of roots colonized, the structure of the community of AM fungi in roots can be assessed using molecular assays, e.g., [29]; biochemical assays, e.g., [30]; phospholipid fatty acid signatures, e.g., [31]; and morphological traits, e.g., [32]. Each of these approaches quantifies aspects of the status of the community of AM fungi at a point in time, but as colonization is dynamic, assessment at more than one time will be more informative in terms of mycorrhizal function [19].

This glasshouse experiment was conducted to evaluate the effect of fertilizers of different elemental P solubility and a microbial inoculant on the colonization of roots by a naturally occurring community of AM fungi in a pasture for two common pasture plants that differ in root architecture. While it is commonly shown that the addition of P fertilizer can decrease the proportion of roots colonized by AM fungi, it is possible that plants with a more extensive root system (such as a grass) may have a lower proportion of colonized roots but a greater length of colonized root [19]. Hence, even a low level of mycorrhizal colonization (%) could correspond to a high mycorrhizal mass in the soil, depending on the plant species. Consequently, an assessment of the proportion of root length colonized may not capture important quantitative differences in mycorrhizal colonization in mixed-species pasture systems. Therefore, the primary focus of this experiment was to investigate potential implications of fertilizer and microbial inoculant treatments on the colonization of roots of grass and legume pasture species by naturally occurring communities of AM fungi in agricultural soils. The extent to which mycorrhizal fungi with different traits might respond to the fertilizers and microbial inoculant used in this study is not known and therefore, this was also investigated.

The specific hypotheses were as follows:

(i) The application of the chemical fertilizer will decrease both the proportion and length of root colonized by AM fungi during the early stages of plant growth, but the grass will have a greater length of colonized root than the legume.
(ii) The application of the rock mineral fertilizer applied at an equivalent P rate to the chemical fertilizer will not affect the proportion or length of AM fungal colonization to the same effect as the chemical fertilizer during the early stages of plant growth.
(iii) The application of the microbial inoculant will stimulate root growth and reduce the colonization of roots by AM fungi.
(iv) The soil treatments (two P fertilizers and a microbial inoculant) will have different effects on the relative abundance of the dominant groups of AM fungi in roots of pasture species which differ in root architecture.

## 2. Materials and Methods

A glasshouse experiment was conducted using a pasture soil from the UWA Farm Ridgefield near Pingelly, Western Australia [18]. Sieved field soil (2 mm) from the pasture was potted (2 kg per pot) as described by Mickan et al. [18]. There were three treatments: (i) a rock mineral fertilizer applied at 75 kg ha^−1^ (~5.6 P kg ha^−1^), (ii) a chemical fertilizer applied at 43 kg ha^−1^ (~5.6 P kg ha^−1^) and (iii) a microbial inoculant. There was an unfertilized control. No other fertilizers were applied to the agricultural soil, including the control. The fertilizers and microbial inoculant were applied prior to sowing the seeds. There were four replicates of each treatment with two harvest times of 5 and 10 weeks after sowing.

The initial characteristics of the soil used in this experiment were 13.6% clay, 12% silt, 76% sand, pH 5.45 in H_2_O, pH 4.93 in CaCl_2_ and electrical conductivity (EC) 163 μS cm^−1^. The available nutrients were Colwell P 65.8 mg kg^−1^ soil, NO_3_^−^ 1.8 mg kg^−1^ soil and NH_4_^+^ 19.52 mg kg^−1^ soil. Bulk density was 1.24 g cm^3^.

The rock mineral fertilizer (MnF) and chemical fertilizer (CF) differed in P content (7.5 and 13.1%, respectively [18]). The solubility of P in CF was 821 mg kg^−1^ P and the solubility of P in MnF was 657 mg kg^−1^ P. The rock mineral fertilizer consisted of fine mineral ores [18]. The chemical fertilizer was Gusto Gold (Summit Fertilizers Australia, Albany, WA, Australia). The microbial inoculant was a combination of bacteria and fungi as stated by Mickan et al. [18].

Subterranean clover (*Trifolium subterraneum* L. cv. ‘Dalkeith’) and Wimmera ryegrass (*Lolium rigidum* Gaudin (1811)) were sown in separate pots. Pots were maintained in a glasshouse under ambient light with a photoperiod of 16/8 h and a temperature of 22/17 C (day/night), and watered by weighing at 70% of field capacity. The soil and roots were sampled, assessed and analyzed according to Mickan et al. [18].

Root colonization by AM fungi was assessed in sub-samples of fresh roots (approximately ~0.5 g fresh weight) cut into 1 cm lengths, cleared in 10% KOH, stained with Trypan blue (0.05%) in lactoglycerol (1:1:1.2) lactic acid:glycerol:water and destained in lactoglycerol [33,34]. The percentage colonization of roots by AM fungi was estimated by visual observation of stained root segments mounted in lactoglycerol by the grid-line intersection counting method which is also used for assessing total root length [35]. The length of root colonized was assessed simultaneously [34] after estimating root length [36].

Root sub-samples (~0.5 g) from the second harvest (10 weeks) were used to extract DNA to assess the AM fungal community composition and relative abundance of dominant AM fungal families [37]. The AMV4.5NF–AMDGR primer target was used and further microbiome analysis was carried out using the SILVIA database. The extraction and DNA sequencing was conducted by the Australian Genome Research Facility (AGRF Ltd., Melbourne, VIC, Australia).

At each harvest, soil nutrient availability (N and P) and soil acidity were assessed as described previously [18]. Total N and P from soil and plant tissue were determined using a Kjeldahl digest [38]. Total N was determined using an ammonium N in green method Na Nitroprusside [39], and total P was calculated using molybdenum blue colorimetry [40].

ANOVA (two-way design) tables (GenStat V.12.1) were used to compare treatment effects for subterranean clover and annual ryegrass dry weight, nutrient concentration, AM colonization (%) and length of root colonized (m). Treatment means were compared using the least significant differences (LSD). A nonmetric multidimensional scaling (NMDS) plot was used to visualize community separation by treatments, and an additional PERMANOVA was used to statistically assess the beta-diversity of the experimental treatments using the Adonis function, as well as alpha diversity indices calculated in the R environment using Vegan, R version 3.4.3 (R Core Development Team, Vienna, Austria, 2017), Vegan version 2.3.0 [41] and GenStat V.12.1.

## 3. Results

### 3.1. Plant Biomass

**Figure 1:**
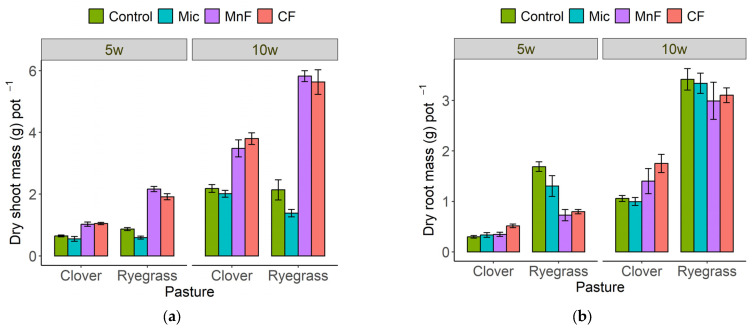
Shoot (**a**) and root (**b**) dry mass (g pot^−1^) for subterranean clover and annual ryegrass grown separately at 5 and 10 weeks. Treatments were untreated soil (control), rock mineral fertilizer (MF), chemical fertilizer (CF) and microbial inoculant (Mic). Data for 10 week harvest previously reported by Mickan et al. [18]. Results are presented as means + standard errors.

At 5 weeks, all treatments significantly influenced shoot and root biomass and there was an interaction between fertilizer and pasture plant treatments (*p* ≤ 0.001) (Table 1). The application of the two fertilizers resulted in a greater increase in shoot mass for annual ryegrass than for subterranean clover, but the microbial inoculant decreased shoot mass for both pasture plants (Figure 1a).

For both subterranean clover and annual ryegrass, the soil P concentration after 5 and 10 weeks was similar for both the rock mineral and chemical fertilizers and greater than for the microbial inoculant and the control (Table 2 and Table 3). The P concentration in soil after application of the microbial inoculant was not different from the control at either harvest times for both plant species (Table 2 and Table 3).

At 5 weeks, the concentration of NO_3_^−^ in soil following the growth of subterranean clover and annual ryegrass was highest for the rock mineral fertilizer treatment (56.1 and 13.6 mg kg^−1^ soil, respectively; Table 2). At 10 weeks, the highest concentration of NO_3_^−^ in soil was also recorded for the rock mineral fertilizer treatment for subterranean clover (23.4 mg kg^−1^ soil) which was greater than for annual ryegrass (6.2 mg kg^−1^ soil), but there was no difference between treatments for annual ryegrass (Table 2 and Table 3). The concentration of NH_4_^+^ in soil for both subterranean clover and annual ryegrass was lower than for NO_3_^−^ with little effect of treatments at either harvest Table 2 and Table 3).

At 5 weeks, the rock mineral and chemical fertilizers and the two pasture species had a significant effect on soil pH, but there was no interaction between them (Table 3). Soil pH decreased significantly following the growth of subterranean clover and annual ryegrass in soil which had received the chemical and rock mineral fertilizers but not the microbial inoculant.

**Figure 2:**
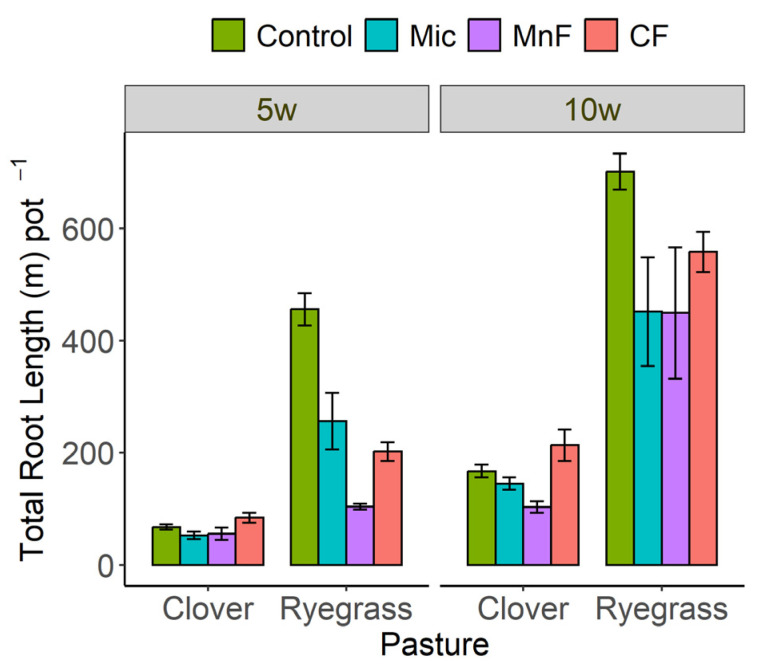
Root length (m pot^−1^) for subterranean clover and annual ryegrass grown separately at 5 and 10 weeks. Treatments were untreated soil (control), rock mineral fertilizer (MF), chemical fertilizer (CF) and microbial inoculant (Mic). Results are presented as means + standard errors.

At 5 weeks, the chemical fertilizer increased both root mass and root length for subterranean clover but there were no effects of other treatments on root mass for this legume (Figure 1b and Figure 2). In contrast, annual ryegrass root mass and root length decreased significantly with the application of all treatments at 5 weeks (Figure 1b and Figure 2).

At 10 weeks, the shoot mass of the subterranean clover increased following the application of the chemical and rock mineral fertilizers, but it was not affected by the microbial inoculant (Figure 1a). The shoot mass of the annual ryegrass also increased following the application of both the chemical and rock mineral fertilizers at 10 weeks, but it decreased with the application of the microbial inoculant (Figure 1a).

At 10 weeks, both fertilizers increased the root mass of the subterranean clover but the microbial inoculant had no effect (Figure 1b). The root length of the subterranean clover was slightly increased only by the chemical fertilizer (Figure 2). In contrast, the annual ryegrass root mass was not affected by any treatments at 10 weeks, but the root length was somewhat reduced by all soil treatments (Figure 1b and Figure 2).

**Figure 3:**
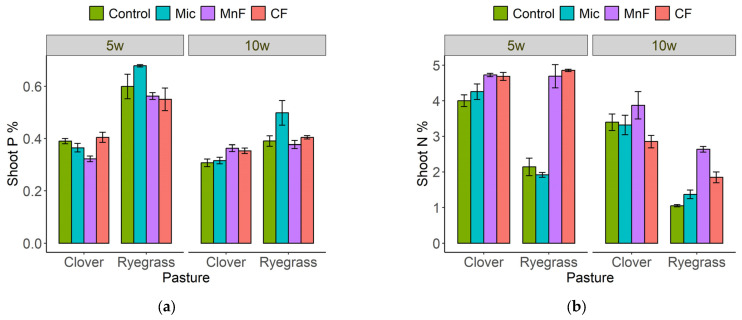
Shoot P (%) (**a**) and shoot N (%) (**b**) for subterranean clover and annual ryegrass grown separately at 5 and 10 weeks. Treatments were untreated soil (control), rock mineral fertilizer (MF), chemical fertilizer (CF) and microbial inoculant (Mic). Results are presented as means + standard errors.

At 5 weeks, shoot P concentration was higher for the annual ryegrass than for the subterranean clover (Figure 3a). The highest shoot P concentration was recorded for the ryegrass for the microbial inoculant treatment at both 5 and 10 weeks (Figure 3a).

Shoot N concentration in subterranean clover was generally unaffected by soil treatments, but for annual ryegrass, both fertilizers increased shoot N concentration at 5 and 10 weeks (Figure 3b).

### 3.2. Mycorrhizal Colonization of Roots

At both 5 and 10 weeks, the form of fertilizer affected mycorrhizal colonization assessed as both the proportion of the root system colonized and the length of root colonized for both subterranean clover and annual ryegrass (Table 1).

**Figure 4:**
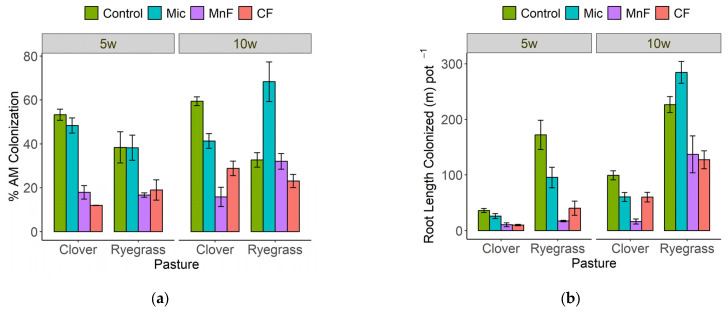
Mycorrhizal colonization as (**a**) % root length colonized and (**b**) length of root colonized (m pot^−1^) for subterranean clover and annual ryegrass grown separately at 5 and 10 weeks. Treatments were untreated soil (Control), rock mineral fertilizer (MF), chemical fertilizer (CF) and microbial inoculant (Mic). Results are presented as means + standard errors.

At 5 weeks, both fertilizers decreased the proportion of roots colonized and the length of root colonized for both subterranean clover and annual ryegrass (Figure 4a,b). The microbial inoculant did not affect either the proportion of roots colonized or the length of root colonized for either plant (Figure 4a,b).

At 10 weeks, both fertilizers and the microbial inoculant decreased the proportion of roots colonized and the length of root colonized for subterranean clover but the decrease was greater for the rock mineral fertilizer, and less so for the microbial inoculant (Figure 4a,b). For annual ryegrass at 10 weeks, only the chemical fertilizer reduced the proportion of roots colonized but both fertilizers decreased the length of root colonized (Figure 4a,b). In contrast with application of microbial inoculant, there was an increase in both the proportion and length of annual ryegrass roots colonized (Figure 4a,b).

### 3.3. AM Fungal Community Composition and Structure

**Figure 5:**
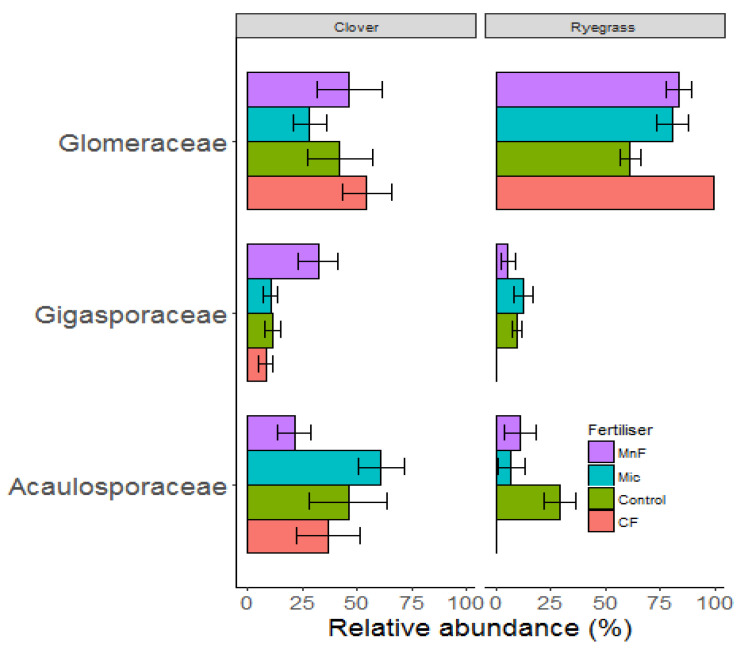
Relative abundance of mycorrhizal families for soils amended with a chemical fertilizer (CF), a rock mineral fertilizer (MnF), a microbial inoculant (Mic) and for an untreated soil (control) in the presence of subterranean clover (**Left**) and annual ryegrass (**Right**) at 10 weeks. Results are presented as means + standard errors.

Three AM fungal families (Acaulosporaceae, Gigasporaceae and Glomeraceae) were represented in the sequencing data set (Figure 5). All three families were influenced by the plant species (*p* < 0.01) and but only Gigasporaceae differed between the fertilizer treatments (*p* < 0.03) (Table 4).

For subterranean clover roots, the rock mineral fertilizer significantly increased the relative abundance of Gigasporaceae, while the chemical fertilizer significantly decreased its relative abundance (Figure 5). Both the rock mineral and chemical fertilizers decreased the relative abundance of Gigasporaceae in roots of annual ryegrass, but this negative effect was greater for the chemical fertilizer. Fertilizer treatments did not affect the relative abundance of either Glomeraceae or Acaulosporaceae but the responses differed between the two pasture species. Glomeraceae was most dominant in roots of annual ryegrass for all the treatments and Acaulosporaceae was more dominant in roots of subterranean clover (Figure 5).

**Figure 6:**
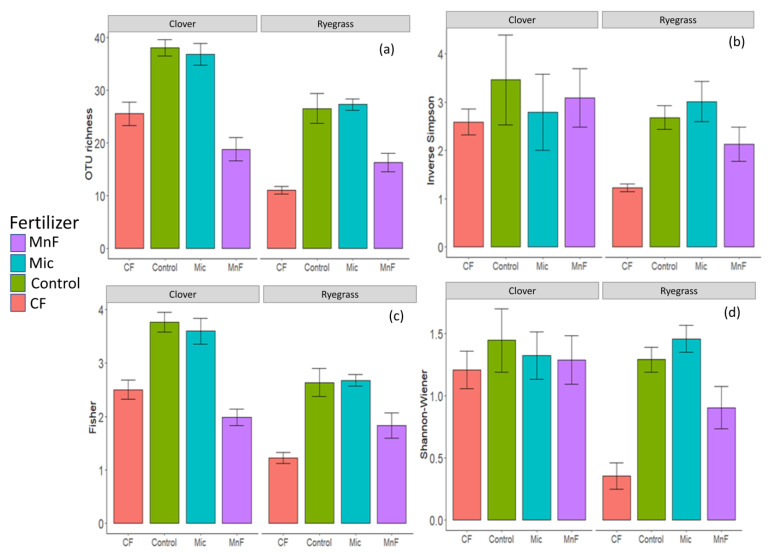
AM fungal community richness and diversity estimators: (**a**) observed OTU richness, (**b**) inverse Simpson index, (**c**) Fisher index and (**d**) Shannon–Wiener index for DNA fungal sequences from subterranean clover and annual ryegrass roots at 10 weeks. Treatments are chemical fertilizer (CF), rock mineral fertilizer (MnF), microbial inoculant (Mic) and untreated soil (control). Bars represent the mean for each treatment; error bars are the standard error.

The rock mineral and chemical fertilizer treatments significantly reduced the richness of the AM fungal OTUs compared to the untreated control (Table 5; Figure 6). However, the diversity of the AM fungal communities in subterranean clover and annual ryegrass differed. For the subterranean clover, the greatest negative effect on AM fungal OTU richness occurred for the rock mineral fertilizer but for the annual ryegrass, the AM fungi OTU richness was the most negative for the chemical fertilizer.

For the subterranean clover, neither of the fertilizers had any effect on the inverse Simpson–Fisher diversity and Shannon–Wiener diversity indices for AM fungi (Table 5; Figure 6). However, the Fisher index was reduced by the chemical fertilizer and the microbial inoculant (Table 3; Figure 6). For the annual ryegrass, the chemical fertilizer had a major effect on the inverse Simpson–Fisher, Shannon–Wiener and Fisher indices of AM fungi (Table 5; Figure 6). The Fisher index was reduced by the rock mineral fertilizer to a lesser extent than the chemical fertilizer and the microbial inoculant was unaffected for annual ryegrass (Table 5; Figure 6).

**Figure 7:**
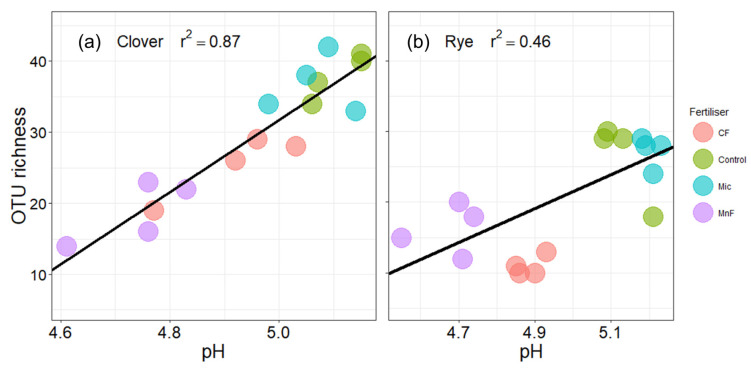
Mycorrhizal community (at 97% similarity OTU level) richness compared with soil pH for soil amended with a chemical fertilizer (CF), a rock mineral fertilizer (MnF), a microbial inoculant (Mic) and untreated soil (control) for (**a**) subterranean clover and (**b**) annual ryegrass at 10 weeks.

For the subterranean clover, soil pH (in water) was highly correlated with the change in OTU richness of AM fungi (R^2^ = 0.87), while there was a weaker correlation for annual ryegrass (R^2^ = 0.46) (Figure 7).

**Figure 8:**
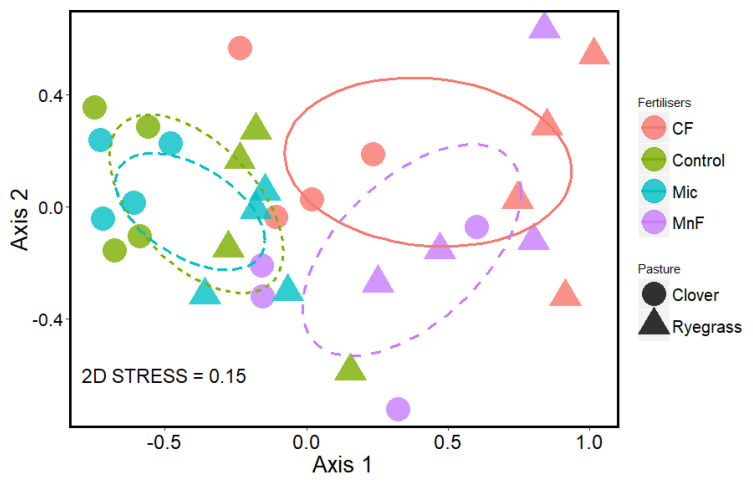
Non-metric multidimensional scaling (NMDS) ordination plot at 97% similarity OTU level, showing differences in mycorrhizal fungal community composition in roots of subterranean clover (circles) and annual ryegrass (triangles) at 10 weeks. The soil treatments were chemical fertilizer (CF), rock mineral fertilizer (MnF), microbial inoculant (Mic) and untreated soil (control). Ellipses represent 95% confidence intervals.

Overall, the AM fungal community composition was strongly affected by fertilizer treatments but not by pasture species. The community composition of the AM fungi in soils amended with the rock mineral and chemical fertilizers differed significantly from those amended with the microbial inoculant and the untreated soil (Figure 8). There was an overlap in the community composition of the AM fungi in response to the application of the rock mineral and chemical fertilizers but the community compositions in the untreated soil and soil treated with the microbial inoculant treatments were similar (Figure 8).

## 4. Discussion

Grasslands cover about 70% of agricultural land globally, but intensive fertilizer use can constrain contributions from soil biological processes in pastures, including those associated with arbuscular mycorrhizal fungi. The contributions that AM fungi have the potential to lead to soil health range from improving fertilizer use efficiency to improving soil health more generally, contributing a level of resilience to soil structure and essentially creating an environment that supports soil biological fertility [42,43]. While these benefits are well known, the extent to which they are expressed by naturally occurring communities of AM fungi in agricultural soils is difficult to quantify [22]. This is primarily because of the dynamic nature of the colonization of roots by different taxa of AM fungi and their plant dependence, especially when compared with the legume root nodule symbiosis which is highly specific and localized on the root [44]. For this reason, we incorporated more than one approach to quantifying AM fungi and investigated the implications of root growth on mycorrhiza formation in pasture plants that commonly grow together in annual pastures in the Mediterranean environment of south-western Australia [45].

According to the first hypothesis investigated, it was expected that the application of the chemical fertilizer would decrease both the proportion and length of root colonized by AM fungi during the early stages of plant growth, but that the grass would have a greater length of colonized root than the legume. Indeed, the proportion of both subterranean clover and annual ryegrass roots colonized did decrease in reponse to application of the chemical fertilizer, with colonization levels decreased to a similar extent for both plants at 5 weeks. However, by 10 weeks, although the length of root colonized was reduced by the chemical fertilizer for both plants, there was a much greater length of mycorrhizal annual ryegrass root present in the soil than for subterannean clover. Consequently, for investigations of the application of fertilizer on mixed grass and legume species, quantification of the percentage of root length colonized alone would not fully capture differences in the impact of chemical fertilizer on a grass mycorrhizal root system compared with a legume mycorrhizal root system [28].

The second hypothesis was that the rock mineral fertilizer applied at an equivalent P rate to that in the rock mineral fertilizer would not affect the proportion or length of root colonized by AM fungal to the same effect as the chemical fertilizer during the early stages of plant growth. However, this was not the case as both fertilizers had a similar effect on decreasing both the proportion and length of root colonized by both plant species. Therefore, the response observed was most likely not a reflection of the form of P, but of the amount of P released by the fertilizers [2].

The third hypothesis was not supported. It was expected that the microbial inoculant would stimulate root growth [46] and that this would lead to a reduction in the length of root colonized by AM fungi. Although the microbial inoculant did not stimulate the root growth of either pasture plant, the length (not the proportion) of root length colonized by AM fungi in annual ryegrass was increased. These differences between plant species appear complex and require further investigation. The complexity of root and AM fungal dynamics make it challenging to estimate mycorrhizal benefits [47,48].

The final hypothesis was that soil treatments (the two P fertilizers and microbial inoculant) would have different effects on the relative abundance of dominant groups of AM fungi in roots of pasture species which differ in root architecture. It was expected that the two P fertilizers and the microbial inoculant would alter the relative abundance of AM fungi in roots of each of the pasture plants in different ways depending on how competitive they were in accessing carbohydrate from roots [49] in addition to direct effects of P [50]. The fertilizers primarily affected the relative abundance of Gigasporaceae. This is likely to be due to the sensitivity of Gigasporaceae to high soil P concentrations [50,51,52] compared to Acaulosporaceae and Glomeraceae [53,54]. Variations in fungal sensitivity to P may be due to differences in requirements of soluble carbohydrates [55], and P can play a crucial role in reducing the concentration of soluble carbohydrates in root exudates [56,57].

The negative effect of fertilizers on AM fungal richness may be due either to (i) competition for carbon allocated by the plant where the dominant fungi should be more competitive [51,55,58], or to (ii) increasing soil nutrient availability that promotes dominance of certain AM fungi [59]. However, the reduction in the proportion of roots colonized by AM fungi with both fertilizers corresponded to reduced soil pH, and under these more acid soil conditions, there was lower OTU richness of AM fungi. Therefore, although phosphorus–carbohydrate interactions may influence the extent of root colonization by AM fungi, associated changes in soil pH can independently influence the diversity of the AM fungal community in the root system. Therefore, with the application of P fertilizers used in our study, changes in soil P and soil pH could simultaneously influence the functioning of AM fungi either directly or indirectly.

## 5. Conclusions

Mixed legume and grass pastures provide complex and dynamic root and rhizosphere habitats for AM fungi. The addition of P fertilizers that differ in solubility when applied at an equivalent level could have significant effects on mycorrhizal communities via effects on root growth parameters. In this study, the traditional P and rock mineral fertilizers had similar effects on the mycorrhizal colonization in both a grass (annual ryegrass) and a legume (subterranean clover). Nevertheless, due to the more extensive root system of annual ryegrass, the impact of the fertilizers in decreasing mycorrhizal root length in the soil would be less for a ryegrass-dominated system than for one dominated by subterranean clover. The greater the length of root colonized, the greater the potential for hyphae to proliferate in soil. This is an important consideration because the contributions that mycorrhizal fungi make to soil aggregation and soil carbon sequestration depend on the total mass of mycorrhizal hyphae in the soil whereas their contribution to plant nutrient and water uptake would be more closely related to the proportion of roots colonized with their associated soil hyphae. Consequently, the complete suite of mycorrhizal benefits cannot be easily predicted from one-off assessments of the proportion of roots colonized or even from assessments of the diversity of the AM fungi in the soil. Furthermore, the reduction in the proportion of roots colonized by the AM fungi in this pasture soil was not directly linked to differences in the relative solubility of P in the fertilizers. The fertilizers reduced soil pH and this was correlated with a reduction in the alpha diversity of the AM fungi in the roots. Thus, the naturally occurring AM fungi in this agricultural soil have the potential to influence the efficacy of use of P fertilizers, but the response would depend on the structure of the pasture plant community, such as the relative abundance of legumes and grasses, and on complex interactions between their roots and the functional traits of the resident AM fungal community.

## Figures and Tables

**Table 1:** ANOVA for shoot dry weight (g pot^−1^), root dry weight, root colonization (%), root length colonized (RLC (m pot^−1^), shoot P% and shoot N% at 5 and 10 weeks after sowing. LSD value given if *p* ≤ 0.05. NS = not significant.

	Shoot Dry Weight		Root Dry Weight		Root Colonization %		Root Length Colonized RLC		Shoot P%		Shoot N%	
ANOVA	*p* Value	LSD	*p* Value	LSD	*p* Value	LSD	*p* Value	LSD	*p* Value	LSD	*p* Value	LSD
**5 weeks**												
Fertilizers	<0.001	0.136	<0.001	0.196	<0.001	8.4	<0.001	2569	0.033	0.05	<0.001	0.38
Pasture	<0.001	0.096	<0.001	0.138	0.11	NS	<0.001	1817	<0.001	0.04	0.771	NS
Fertilizers × Pasture	<0.001	0.192	<0.001	0.277	0.06	NS	<0.001	3634	0.023	0.07	<0.001	0.53
**10 weeks**												
Fertilizers	<0.001	0.49	0.604	NS	<0.001	9.2	<0.001	3429	0.075	NS	<0.001	0.43
Pasture	<0.001	0.35	<0.001	0.3	0.403	NS	<0.001	2425	<0.001	0.031	<0.001	0.31
Fertilizers × Pasture	<0.001	0.69	0.045	0.61	<0.001	13.1	<0.001	4849	0.004	0.061	<0.013	0.61

**Table 2:** Soil available nitrate, ammonium and extractable Colwell P for subterranean clover and annual ryegrass after 5 and 10 weeks for treatments C (control), CF (chemical fertilizer), Mic (microbial inoculant, and MnF (rock mineral fertilizer). Parameter means with the same letter are not significantly different among treatments (*p* > 0.05) according to the Tukey test. Results of the two-way ANOVA are indicated for each parameter.

Treatment	NO_3_^−^ mg kg^−1^ Soil				NH_4_^+^ mg kg^−1^ Soil				Colwell P Mg kg^−1^ Soil			
	Clover		Ryegrass		Clover		Ryegrass		Clover		Ryegrass	
	5 w	10 w	5 w	10 w	5 w	10 w	5 w	10 w	5 w	10 w	5 w	10 w
C	13.92 b	6.96 ab	0.14 a	6.26 a	0.76 a	0.77 a	0.44 a	0.81 a	141 a	135 a	131 a	1311
CF	32.14 c	9.39 b	1.77 a	5.76 a	1.19 b	0.80 a	0.66 a	0.78 a	189 c	160 bcd	169 bc	185 d
Mic	3.14 a	6.18 a	0.41 a	5.81 a	0.54 a	0.82 a	0.49 a	0.83 a	148 ab	144 abc	134 a	137 ab
MnF	56.18 d	23.43 c	13.61 b	6.27 a	1.52 b	1.14 b	1.31 b	0.77 a	169 bc	170 cd	160 bc	174 d

**Table 3:** ANOVA for available nitrate, ammonium and extractable Colwell P and soil pH (water and CaCl_2_) for subterranean clover and annual ryegrass after 5 and 10 weeks after sowing. LSD value given if *p* ≤ 0.05. NS = not significant.

	NO_3_^−^		NH_4_^+^		P		pH Water		pH CaCl_2_	
ANOVA	*p* Value	LSD	*p* Value	LSD	*p* Value	LSD	*p* Value	LSD	*p* Value	LSD
**5 weeks**										
Fertilizers	<0.001	2.72	<0.001	0.16	<0.001	13	<0.001	0.063	<0.001	0.056
Pasture	<0.001	1.93	<0.001	0.11	0.066	NS	<0.001	0.044	0.002	0.040
Fertilizers × Pasture	<0.001	3.85	0.031	0.22	0.563	NS	0.089	NS	0.444	NS
**10 weeks**										
Fertilizers	<0.001	1.21	0.005	0.097	<0.001	11	<0.001	0.073	<0.001	0.063
Pasture	<0.001	0.86	0.010	0.069	0.287	NS	0.572	NS	0.442	NS
Fertilizers × Pasture	<0.001	1.72	<0.001	0.137	0.039	16	0.043	0.104	0.109	NS

**Table 4:** Root AM fungi two-way ANOVA results showing *p* values fixed at family resolution of relative abundance. Treatments consisted of ‘Fertilizer’, and ‘Pasture’. Significant *p* values indicated by * and *** corresponding to *p* < 0.05 and < 0.001, respectively.

AM Fungus Family	Treatment	df	Sum of Squares	Mean Squares	F. Model	Pr (>F)
Acaulosporaceae	Fertilizer	3	2768.2	922.7	2.2087	0.113
	Pasture	1	6953.7	6953.7	16.644	<0.001 ***
	Fertilizer × Pasture	3	2368.7	789.6	1.8899	0.158
	Residuals	24	10,026.5	417.8		
Gigasporaceae	Fertilizer	3	840.27	280.09	3.674	0.026 *
	Pasture	1	634.12	634.12	8.3179	0.008 *
	Fertilizer × Pasture	3	952.27	317.42	4.1637	0.017 *
	Residuals	24	1829.65	76.24		
Glomeraceae	Fertilizer	3	3159.2	1053.1	2.8571	0.058
	Pasture	1	11,788.2	11,788	31.982	<0.001 ***
	Fertilizer × Pasture	3	1237.3	412.4	1.1189	0.361
	Residuals	24	8846.1	368.6		

**Table 5:** Root AM fungi two-way ANOVA results showing *p* values fixed at family resolution of relative abundance. Treatments consisted of ‘Fertilizer’, and ‘Pasture’. Significant *p* values indicated by * and *** corresponding to *p* < 0.05 and < 0.001, respectively.

Taxon	Treatment	Degrees of Freedom	Sum of Squares	Mean Squares	F. Model	Pr (>F)
Shannon	Fertilizer	3	1.95	0.65	5.80	0.004 *
	Pasture	1	0.79	0.79	7.06	0.014 *
	Fertilizer × Pasture	3	1.06	0.35	3.14	0.044 *
	Residuals	24	2.69	0.11		
Inverse Simpson	Fertilizer	3	6.33	2.11	1.83	0.169
	Pasture	1	4.15	4.15	3.59	0.070
	Fertilizer × Pasture	3	2.74	0.91	0.79	0.512
	Residuals	24	27.74	1.16		
Fisher	Fertilizer	3	13.17	4.39	29.63	<0.001 ***
	Pasture	1	6.05	6.05	40.86	<0.001 ***
	Fertilizer × Pasture	3	1.49	0.50	3.35	0.036 *
	Residuals	24	3.56	0.15		
Richness	Fertilizer	3	1627.00	542.33	36.66	<0.001 ***
	Pasture	1	722.00	722.00	48.81	<0.001 ***
	Fertilizer × Pasture	3	156.00	52.00	3.52	0.030 *
	Residuals	24	355.00	14.79		
Eveness	Fertilizer	3	0.11	0.04	4.28	0.015 *
	Pasture	1	0.04	0.04	4.28	0.049 *
	Fertilizer × Pasture	3	0.11	0.04	4.22	0.016 *
	Residuals	24	0.20	0.01		

## Data Availability

The data supporting the results and conclusions in this manuscript are included within the paper, and raw data can be shared with researchers upon request.
